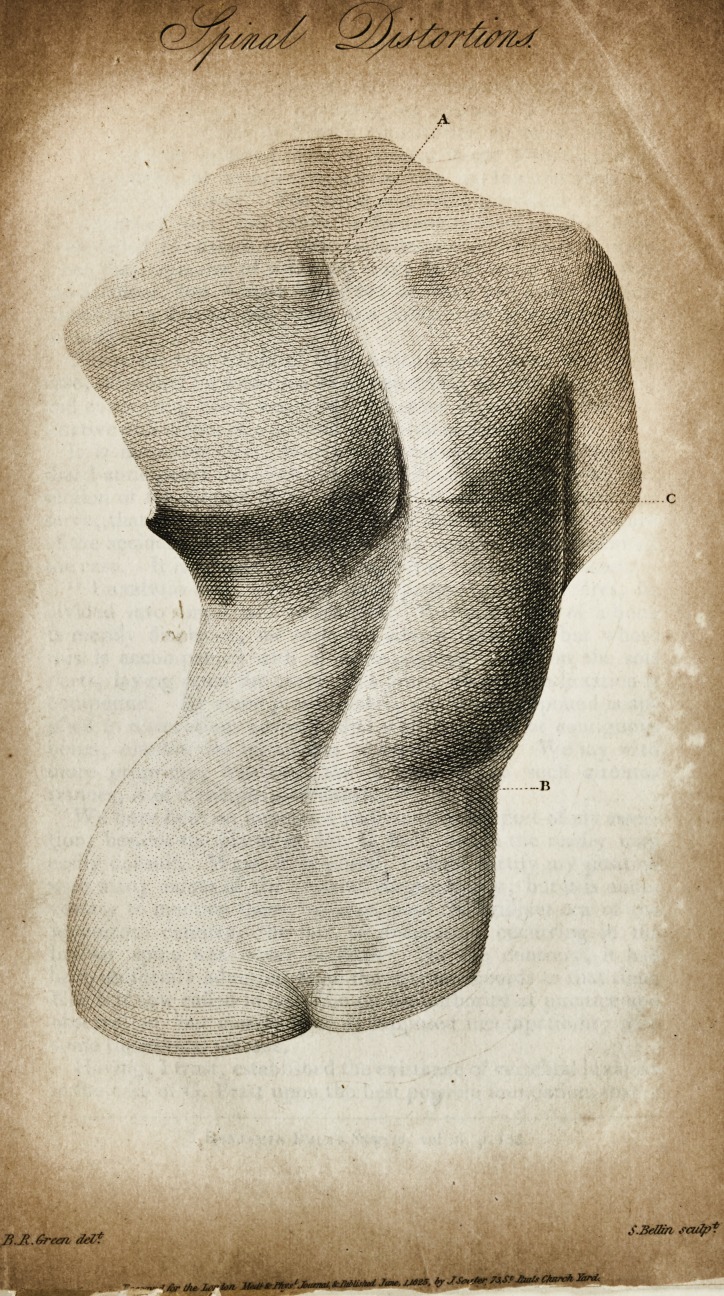# Remarks upon Certain Cases of Spinal Disease, Published in the 302d and 303d Numbers of This Journal

**Published:** 1825-06

**Authors:** E. Harrison


THE LONDON
### Medical and Physical Journal.

No. 6 OF VOL. LIII.] JUNE, 1825. [No. 316.

For many fortunate discoveries in medicine, and for the detection of numerous errors, the world is indebted to the rapid circulation of Monthly Journals; and there never existed any work, to which the Faculty, in Europe and America, were under deeper obligations, than to the Medical and Physical Journal of London, now forming a long, but an invaluable, series.—RUSH.

### ORIGINAL COMMUNICATIONS, SELECT OBSERVATIONS, &c.

ART. I.—*Remarks upon certain Cases of Spinal Disease, published in the 302d and 303d Numbers of this Journal.* By E. HARRISON, M.D. &c. &c.

On turning over the pages of your periodical a few days since, I perceived, in the review of Mr. Bampfield's work, a reference to some of my papers formerly communicated to your Journal. From the nature of the remarks alluded to, I feel myself called upon to explain some of my opinions more at large; and shall, moreover, assert my prior claim to doctrines and modes of practice which have been adopted by some late writers, without referring to my name or pretensions, and therefore, as I conceive, with a design to pass my opinions and practice for their own. This procedure, indefensible at all times, is most reprehensible in persons who freely animadvert upon such other parts of my writings as they deem to be objectionable.

Others, again, will require attention, whose researches have been so exclusively devoted to anatomy as to preclude the opportunity of acquiring clear views upon other subjects. These gentlemen seem to fancy that all medical science lies concealed in the human charnel-house. To the necessity of anatomy in forming the medical character, I most readily assent: no one values it more than I do, and in early life few took more pains to understand the human structure. In pursuit of this object, I voluntarily laboured at practical anatomy in three distinguished capitals. Still it constitutes only one of many pillars in the Esculapian temple. How, for example, would the anatomical physician be able to relieve his patient, without a knowledge of therapeutics and pharmaceutical chemistry? and what advantage could be derived from both, unless he made them subservient to experience and observation?

I shall commence by referring your readers to the case of

Miss D. J.* and describing the plates illustrative thereof: the first shows the curvature before the treatment began; the second is a correct view of the back, after the process described in relating the case had terminated.

A. The upper;
B. The lower part of the spine.
C. Its greatest bend towards the right.

The above-described print was taken from a model in my possession, of which it is a faithful representation. If a straight line be drawn on the model, between the corresponding letters A and B of the print, it would extend an inch and a half below C. The spine, moreover, dips and sinks inwards, or forwards, an inch and three-quarters at the point C. This internal projection, though scarcely discernible in the engraving, is quite apparent on the model. The two incurvations and deviations from the perpendicular are occasioned by a double flexion of the backbone; one lateral, the other internal, or anterior. Both curvatures are so considerable, that we can only explain them (as it strikes me) by admitting a two-fold departure, or removal, of the spinal column out of its natural situation in the back. I cannot agree with Dr. Dods, that these are simple contortions or rotations of the vertebræ. Much more is required to explain the changes shown on the model. In my judgment, they proceed from nothing less than a real alteration in the position and location of the vertebræ themselves. On no other supposition can I understand how this extensive and complicated distortion could have arrived at its present alarming height. We have further to remark, that the right scapula rises two inches above the dip or hollow, and is more elevated, by an inch, than the left. Taking it for granted that the spine had actually moved out of its original place in the back to the distances mentioned, we have yet to inquire by what agency these extraordinary removals were effected. Had the case above described been only a solitary instance of the kind, it would have afforded greater scope for speculation and controversy; other similar examples might have been called for to establish the fact. Happily, no such difficulty presents itself on this occasion. Numerous lateral and protuberant gibbosities, of equal magnitude, may be seen in my collection; some of which will soon be communicated to the public. In the mean time, I wish to refer my readers, should they be desirous of additional testimonies, to the histories and prints† explanatory of the diseased state of the spine in Mr. George Andrews and Miss Tarrant. In neither of these cases had the vertebræ protruded strictly backwards; there is a little obliquity in both; nor have I met with a single deformity

* See this Journal, vol. 51. † Ibid.

where the vertebræ were driven immediately outwards or sideways. There is always deviation, and this varies considerably in different protuberances. Sometimes the curvature is almost exactly backwards; at other times it is nearly lateral; but never entirely either one or the other. My attention was forcibly drawn to this circumstance at an early period of my inquiries, and I have had them confirmed on so many occasions, that I have long entertained an opinion that the projecting vertebræ are constantly extruded in a slanting direction. This necessarily gives them a twist, or rotatory movement. Dr. Dods, perceiving this deviation in the appearance of the projecting vertebræ, was led to believe that it proceeded from some irregular muscular action; whereas the change is wholly occasioned by the manner of their presentation. I have accordingly found it sometimes much more considerable than at others. Whatever may be the true cause of distortion, it appears to me that the affected vertebræ assume an oblique direction, because they are constrained equally by the spinous and transverse processes, with their ligamentary connexions, from protruding immediately backwards or sideways.

Having, I think, proved that a simple twist, or partial turn of the vertebræ, could not have depressed the column a whole inch in Miss D. J.'s case, or thrust it sideways a full inch and a half out of the line, some other cause, besides rotation or contortion, must be looked for, to explain the incurvations with which this interesting patient was afflicted. Should Dr. Dods, or the other advocates for muscular action, endeavour to reconcile this deformity with their favourite hypothesis, I wish to admonish them, that the five last cases published by me in your Journal are of the same description, and require explanation before their proofs will be deemed complete. Hitherto no one has condescended to fix upon the particular muscles supposed to be affected, and to show by what irregular movements the malady is produced. Dr. Dods boldly asserts that nearly three hundred muscles are so engaged: on what authority I know not, as I cannot, for obvious reasons, enter into his calculation.

In referring to the case of G. Pratt, first introduced into your Journal December 1820, you omitted to notice my subsequent remarks upon the same patient, inserted in your Number for May 1824. As this case is of importance to illustrate some disputed points in spinal pathology, and has given rise to cavil and misrepresentation, I beg leave to restate some of the leading particulars, for the consideration of your numerous readers. "On examination, I found the first lumbar vertebra wholly dislocated and driven into the left loin. The right transverse process is sunk downwards; the opposite one has risen, and can be distinctly felt below the skin. The last dorsal and second lumbar vertebræ were also displaced by the accident. Several other dorsal bones are gradually suffering spontaneous luxation, for want of vertebral support below them."*—(*Sept. 22d*, 1820.)

"This dislocation was of the species described by Celsus, in which the vertebræ 'toto loco motæ sunt.' I only saw the boy twice. He was brought to me the first time. On calling a few days afterwards, I recommended to his father an inspection of the diseased parts, when opportunity offered; from which it is to be inferred, that I thought something more would be disclosed by a post-mortem investigation. I also declared that, together with dislocation, some of the ligaments would be found broken, and the nervous substance injured. I said nothing about fracture of the vertebræ, as has been erroneously asserted. The tract of the lumbar spine lies so deep, that, after a lapse of nine months, it would, I conceive, have been next to impossible to come to a satisfactory decision upon such a question. Here terminated my connexion with the patient. I might have justly laid myself open to the imputation of ignorance and presumption, had I made any attempt, under these hazardous circumstances, to remove the luxation. Such was my conviction of its severity and fatal tendency at the first interview, that I positively refused, both then and afterwards, to interfere professionally, or attempt any thing for its restoration, though urgently importuned by the mother to afford my assistance."†

The child, it seems, survived the accident thirteen months, and died of croup. Mr. Charles Bell has preserved the morbid parts, and favoured us with two engravings to represent the mischief inflicted upon the spine and cord. He observed to his pupils, on producing these specimens, "The *dislocation*, you perceive, is complete."‡ I have not only given a direct reference to Mr. Charles Bell's report, (with which mine, made during life, wholly corresponds,) but have inserted his very words, to show that my previous statement of the case, notwithstanding all that has been said and written, is fully confirmed by the dissections of this able anatomist. His first plate represents the luxated vertebra, with the connecting ligament placed between it and the last dorsal; his second displays the torn extremities, and disorganised condition of the spinal marrow at the injured part. The dissection and plates fully confirm both my statements, as will appear by a reference to them.

They prove, it is true, that greater mischief was inflicted upon the luxated joint than I had mentioned in the report made during life: nor could it be expected, after an interval of nine months, that a deep-seated fracture would be apparent and distinguishable in the unnatural position of the extruded

* *London Med. and Phys. Journal*, vol. 44. † Ibid. vol. 51.

‡ See Bell's *Observations on Diseases of the Spine*, p. 25, 79.

### Spinal Distortions.

B. R. Green del.t S. Bellin sculp.t

Engraved for the London Med.l & Phys.l Journal, & Published, June, 1, 1825, by J. Souter, 73, St. Pauls Church Yard.

### *Spinal Distortions.*

*B. R. Green del.t* *S. Bellin sculp.t*

[illegible] for the London Med. & Phys.l Journal, & Published June, 1 1825, by J. Souter, 73, St Pauls Church Yard.

vertebra. In that long period, nature was busily employed in repairing the injury and obviating its effects. The powerful and continued efforts of Pratt's constitution to overcome the severe mischief inflicted upon it, appear in the formation of a new ligament, or membrane, connecting the luxated vertebra with the last dorsal. The same admirable provision to avert the effects of hurts and blows is frequently seen in what have been called accidental or false articulations. These take place when the fragments of a broken bone do not unite again after the accident, but remain movable; or when the bones, which constitute a diarthrodial joint, are completely severed and disunited.

There are two kinds of false articulations: one, as in this case, is formed by a fibrous band extending to both fragments, and connecting them together; the other displays all the distinctive characters of a perfect diarthrodial joint.

It is moreover clear, from my conversation with the father, that I anticipated the discovery of additional mischief from dissection of the morbid parts. And here it will be proper to observe, that the greater extent of injury does not alter the nature of the accident, or militate in the least against my opinion of the case. It is equally dislocation, but of a mixed character.

"Luxations may, with the same propriety as fractures, be divided into simple and compound. Where the end of a bone is merely displaced, we term it a simple luxation; but where this is accompanied with a corresponding wound in the soft parts, laying open the cavity of a joint, we say the luxation is compound. By some practitioners, the term compound is applied to dislocations accompanied with fractures of contiguous bones, whether the teguments be injured or not. We say with more propriety, however, that a luxation, in such circumstances, is of a complicated nature."*

We have here an unbiassed testimony in support of my assertion, besides the plates of Mr. C. Bell, which the reader may easily consult. Were it required, I could fortify my position with many names of the greatest respectability; but it is unnecessary to mention them, because, until the brilliant era of the nineteenth century, the fact of dislocation occurring in the lumbar spine was never doubted! On the contrary, it had been uniformly admitted from the earliest records to that time. Nor is it now called in question on the authority of practice and observation, but merely from its supposed incompatibility with some theoretical notions.

Having, I trust, established the existence of vertebral luxation in the case of G. Pratt upon the best possible foundation, that of

---

* Benjamin Bell's *Surgery*, vol. vi. p. 158.

uniform experience, I shall resume the inquiry in another place, to show that dislocations in the vertebræ may be complicated with caries, with softness of the bones, and exuberant ossification, as well as with fractures of the vertebræ. This is all that I designed to express by the first dislocation, and the spontaneous luxation, or subluxation, which followed in some other vertebræ. The latter term is, perhaps, to be preferred in this place, because it has a more limited meaning than the former, which includes every gradation, from the slightest parting to entire disunion. The interpretation now given is agreeable to strict etymology, and accords with the best ancient and modern descriptions of this most important, though often mistaken, form of human misery.

In recording Pratt's case, and describing the appearances, I had no intention of confounding it with dislocations induced by constitutional causes. His originated in the sudden application of external force; the latter proceeds from the slow operation of internal means. These two forms differ from one another in so many respects, and require, moreover, such an opposite treatment, that they ought never to be confounded in practice.

*Holles-street, Cavendish-square; March* 15*th*, 1825.

## Figures and Tables

**Figure f1:**
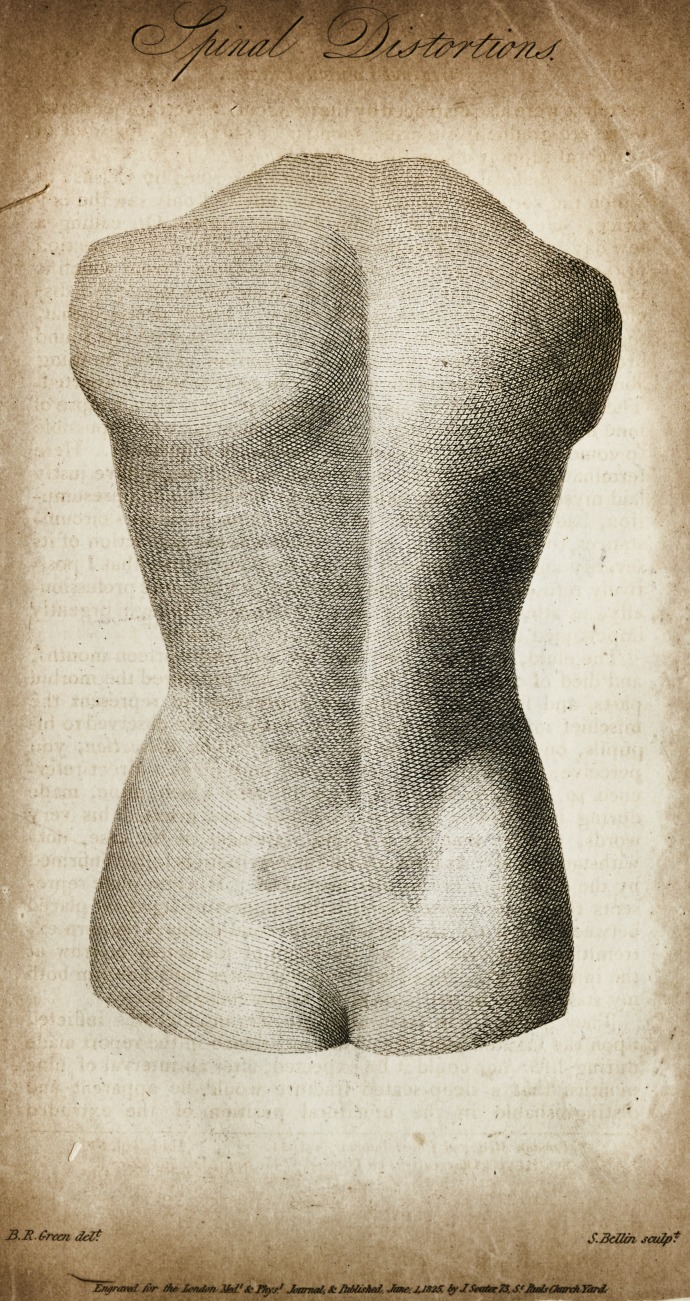


**Figure f2:**